# Molecular and Biological Characterization of the First Hypovirus Identified in *Fusarium oxysporum*

**DOI:** 10.3389/fmicb.2019.03131

**Published:** 2020-01-24

**Authors:** Almudena Torres-Trenas, M. Carmen Cañizares, M. Dolores García-Pedrajas, Encarnación Pérez-Artés

**Affiliations:** ^1^Departamento de Protección de Cultivos, Instituto de Agricultura Sostenible, Consejo Superior de Investigaciones Científicas, Córdoba, Spain; ^2^Instituto de Hortofruticultura Subtropical y Mediterránea “La Mayora”, Universidad de Málaga, Consejo Superior de Investigaciones Científicas, Málaga, Spain

**Keywords:** hypovirus, FodHV2, *Fusarium oxysporum*, *Hypoviridae*, mycovirus

## Abstract

A novel mycovirus named Fusarium oxysporum f. sp. dianthi hypovirus 2 (FodHV2) has been identified infecting isolates *Fod* 408 and *Fod* 409 of *Fusarium oxysporum* f. sp. *dianthi* from Morocco. The genome of FodHV2 is 9,444 nucleotides long excluding the poly(A) tail, and has a single open reading frame encoding a polyprotein. The polyprotein contains three highly conserved domains of UDP glucose/sterol glucosyltransferase, RNA-dependent RNA polymerase, and viral RNA helicase. In addition, particular residues of Cys, Hys, and Gly detected in the N-terminal region suggest the presence of the catalytic site of a highly diverged papain-like protease. Genomic organization, presence of particular conserved motifs, and phylogenetic analyses based on multiple alignments clearly grouped FodHV2 with the members of the family *Hypoviridae*. FodHV2 was transferred by hyphal anastomosis to a recipient Hyg^R^-tagged virus-free strain. The comparison of the infected and non-infected isogenic strains showed that FodHV2 did not alter the vegetative growth, neither the conidiation nor the virulence of its fungal host. Efficiency of FodHV2 transmission through the conidia was 100% in both the original and the recipient infected-isolates. To the best of our knowledge, this is the first report of a hypovirus infecting the plant pathogen *F. oxysporum*, and also the first one of a hypovirus detected in a fungal strain from the African continent.

## Introduction

The soilborne fungal species *Fusarium oxysporum* contains a diversity of host–plant specific forms (*formae speciales*) that cause vascular diseases in a large number of economically important crops. *F. oxysporum* f. sp. *dianthi* is the *forma specialis* that infects carnation, causing the most severe disease in this crop worldwide (Garibaldi and Gullino, 1987; Baayen et al., 1997). The control of vascular wilt of carnation has been tried mainly by applying soil fumigants such as methyl bromide before planting, together with the use of resistant varieties. However, the recent prohibition on the use of methyl bromide, together with the difficulty in obtaining carnation varieties resistant to the different pathogenic variants (races) of the fungus, made control of this disease ineffective. Alternatively, methods based on the addition to the soil of organic amendments such as poultry manure or pepper compost, followed by soil solarization, were developed and applied with variable success (Melero-Vara et al., 2011).

Mycoviruses (viruses that infect fungi) are found in all major groups of phytopathogenic fungi (reviewed in Ghabrial and Suzuki, 2009; Pearson et al., 2009; Son et al., 2015). Most mycoviruses possess either single-stranded or double-stranded RNA genomes. A recent *in silico* study analyzing transcriptome datasets from fungi has revealed an unknown frequency and diversity of viral infections (Gilbert et al., 2019). Viral infections are frequently cryptic, but in some cases an alteration of particular phenotypic fungal traits, including virulence, can be observed. The use of hypovirulence-associated mycoviruses has been proposed as a new strategy for biological control (virocontrol) of fungal diseases (Chiba et al., 2009; Ghabrial and Suzuki, 2009). The most studied case is that of Cryphonectria hypovirus 1 (CHV1), a mycovirus in the family *Hypoviridae* that has been successfully used for the control of chestnut blight caused by *Cryphonectria parasitica* in Europe (Milgroom and Cortesi, 2004; Nuss, 2005). Currently recognized hypoviruses, Crypohonectria hypovirus 1-4 (CHV1, CHV2, CHV3, CHV4), were all isolated from the chestnut blight fungus *C. parasitica* (Fulbright, 1984; Shapira et al., 1991; Hillman et al., 1992, 1994; Smart et al., 1999; Hillman et al., 2000; Yuan and Hillman, 2001; Linder-Basso et al., 2005), and are classified into four homonymous species in the genus *Hypovirus*, family *Hypoviridae*. Success in using CHV1 for the biological control of chetnust blight has prompted interest in identifying new members of this family in other phytopathogenic species. Consequently, a number of novel related viruses were isolated from different phytopathogenic fungi, including Alternaria alternata hypovirus 1 (AaHV1; Li et al., 2019), Botrytis cinerea hypovirus 1 (BcHV1; Hao et al., 2018), Entoleuca Hypovirus 1 (EnHV1; Velasco et al., 2018), Phomopsis longicolla hypovirus 1 (PlHV1; Koloniuk et al., 2014), Rosellinia necatrix hypovirus 1 and 2 (RnHV1 and RnHV2; Arjona-Lopez et al., 2018; Velasco et al., 2018), Sclerotinia sclerotiorum hypovirus 1 and 2 (SsHV1 and SsHV2; Xie et al., 2011; Hu et al., 2014), Setosphaeria turcica hypovirus 1 (StHV1; Gilbert et al., 2019), and Valsa ceratosperma hypovirus 1 (VcHV1; Yaegashi et al., 2012). In the genus *Fusarium*, four putative members of the family *Hypoviridae* have been identified: Fusarium graminearum hypovirus 1 (FgHV1) from *F. graminearum* strain HN10 (Wang et al., 2013), Fusarium graminearum hypovirus 2 (FgHV2) from *F. graminearum* strain SJ16 (Li et al., 2015), Fusarium poae hypovirus 2 (FpHV2) from *F. poae* strain MAFF 240374 (Osaki et al., 2016), and Fusarium langsethiae hypovirus 1 (FlHV1) from *F. langsethiae* strain AH32 (Li et al., 2017). All the above-mentioned viruses have not yet been approved by the International Committee of Taxonomy for Viruses (ICTV) as members of the family *Hypoviridae* (Suzuki et al., 2018).

All recognized hypoviruses are non-encapsidated, positive single-stranded RNA viruses. The genomes of hypoviruses range from 9 to 14 kilobase (kb) in length excluding a poly(A) tail, and possess one or two open reading frames (ORFs) on their coding strands. Genomic sequences of all hypoviruses contain conserved domains of a papain-like protease, an RNA-dependent RNA polymerase (RdRp), and an RNA helicase (Suzuki et al., 2018). Depending on the presence of either one or two ORFs, and also based on genomic characteristics and phylogenetic analyses, the viral family *Hypoviridae* has been recently proposed to be divided into two (“Alphahypovirus” and “Betahypovirus”) (Yaegashi et al., 2012; Khalifa and Pearson, 2014; Li et al., 2015), or three (“Alphahypovirus,” “Betahypovirus,” and “Gammahypovirus”) (Hu et al., 2014) genera.

Despite its denomination, infection by a hypovirus does not always result in the induction of hypovirulence in its fungal host. Virulence levels among different hypoviruses vary considerably. For example, among the four hypoviruses identified infecting different strains of *C. parasitica*, CHV1 and CHV2 alter fungal growth and strongly reduce fungal virulence (Hillman et al., 1990, 1992, 1994). On the other side, CHV3 has little effect on fungal growth while also reduces substantially the virulence of *C. parasitica* (Fulbright, 1984; Smart et al., 1999), and CHV4 has not observable effect on its host (Enebak et al., 1994; Linder-Basso et al., 2005). In the case of hypoviruses identified in *Fusarium* species, only the hypovirus detected in *F. graminearum* isolate SJ16 (FgHV2) has been associated with hypovirulence (Li et al., 2015), whereas FgHV1 does not alter the virulence of its host (Wang et al., 2013), and the possible effect of FlHV1 or FpHV2 has not been reported.

In this work we describe and biologically characterize a putative novel virus in the family *Hypoviridae*, for which we proposed the name Fusarium oxysporum f. sp. dianthi hypovirus 2 (FodHV2). It is the first hypovirus identified in the species *F. oxysporum.*

## Materials and Methods

### Fungal Isolates and Culture Conditions

Strains of *F. oxysporum* f. sp. *dianthi* used in this study were isolated in 2012 from soil samples and diseased carnation plants collected in a carnation-growing area in Morocco (Table 1). All isolates were characterized to race and molecular group using multiplex-PCR, as previously described (Gómez-Lama Cabanás et al., 2012). Isolates were stored at −80°C in glycerol, and propagated on potato dextrose agar (PDA) medium at 25°C in the dark.

**TABLE 1:** *Fusarium oxysporum* f. sp. *dianthi* isolates analyzed.

Isolate (s)^(a)^	Source	Race group assignation by PCR pattern^(b)^
*Fod* 406, 407, 409, 410, 411, 412, 424, 425	Plant	R1t
*Fod* 413, 414, 416, 417, 418, 419, 421,422, 426, 427, 428, 429, 430, 431, 432	Soil	R1t
*Fod* 415, 420, 423	Soil	R2II
*Fod* 408	Plant	R2II

*^(a)^All isolates were from Morocco. ^(b)^Race assignation by multiplex specific-PCR amplifications, as described in Gómez-Lama Cabanás et al. (2012). R1t = race 1 type, R2II = race 2 molecular group II.*

### dsRNA Purification

Viral dsRNA was purified by cellulose column chromatography (Valverde et al., 1990) from the mycelium collected after 7 days of growth in potato dextrose broth, as previously described (Lemus-Minor et al., 2018). The purified dsRNA extracts were analyzed by electrophoresis in 0.8% agarose gels stained with RedSafe^TM^ Nucleic Acid Staining Solution (iNtRON Biotechnology, Seongnam-si, South Korea). The dsRNA nature of the extracts was confirmed by digestion with DNase I and RNase A treatment in low salt condition.

### cDNA Synthesis, Cloning, Sequencing, and Phylogenetic Analysis

The complete sequence of the viral RNA was determined using a combination of techniques as reverse transcription (RT), polymerase chain reaction (PCR) amplifications, cloning and Sanger sequencing. RT and PCR amplifications were performed using random hexamer priming, and amplified products were cloned into the vector pCR Blunt (Invitrogen, Carlsbad, CA, United States) and sequenced. Analysis of these partial nucleotide sequences with the BLASTX algorithm in the NCBI database arranged them according to a high sequence similarity with other mycoviruses. Specific primers were then designed to fill in the sequence gaps. Clones for the terminal sequences of the dsRNA were generated by Single Primer Amplification Technique (SPAT), using T4 RNA ligase oligonucleotide-mediated amplification (Xie et al., 2006). Assembly of these sequences was performed using the software Lasergene SeqMan^TM^ Version 7.0.0 (DNASTAR^®^ Inc., Madison, WI, United States). For comparison of the nucleotide sequences of the 5′- and 3′-UTRs, and the amino acid (aa) sequences of conserved domains, multiple sequence alignments were carried out using the software MAFFT version 7 with the default parameters (Katoh and Standley, 2013). Phylogenetic trees were constructed using the program Tree View of Geneious 8.1.5 package (Biomatters), and generated by the Neighbor-Joining (NJ) method (Saitou and Nei, 1987) with 1000 bootstrap replicates.

### Virus Detection

Infection of fungal isolates with FodHV2 was tested by performing a RT-PCR on denatured dsRNAs using specific primers directed to the RdRp sequence of FodHV2. Primers FodHV2RT (5′-CAGGGACTACAGGTAAAGT-3′) and FodH V2F/FodHV2R (5′-GGAAGTTGGTGGAGGAGCTG-3′/5′-GT ACTTTTGTGCTTCTTCCAGG-3′) were used for the RT and the PCR, respectively. The products of the RT-PCR amplifications were analyzed by electrophoresis in 1.5% agarose gels. All resulting amplicons were purified from the gel, sequenced and analyzed for homology with the sequence of FodHV2 using the program Geneious version 8.1.5 (Biomatters).

### Virus Transmission

To analyze the effect of FodHV2 on its fungal host, two isogenic fungal isolates, infected and not infected with the mycovirus, were compared. We first attempted to obtain the isogenic versions by selecting single conidia from the originally infected isolate *Fod* 408. Selection of the conidia was performed as described (Lemus-Minor et al., 2019), and the dsRNA extracts of the resulting monoconidial cultures were analyzed by cellulose column chromatography to check for the presence of FodHV2 dsRNA. In another approach, FodHV2 was transmitted by hyphal anastomosis from the strain originally infected (*Fod* 408, donor), to another virus-free strain that had been previously transformed with a hygromycin resistance gene (*Fod* 77Hyg^R^, recipient), as described in Lemus-Minor et al. (2019). Presence of FodHV2 in recipient isolate *Fod* 77Hyg^R^ was confirmed by dsRNA extraction and RT-PCR. In addition, efficiency in virus transmission through the spores in the new infected isolate *Fod* 77Hyg^R^ was also determined.

### Effect of FodHV2 on Selected Phenotypic Traits of Its Fungal Host

The effect of FodHV2 on its fungal host was analyzed by determining differences in mycelial growth rate between the virus-free (*Fod* 77Hyg^R^) and the virus infected (*Fod* 77Hyg^R^HV^+^) isolates on solid medium, conidiation rate in liquid medium, and virulence on carnation. To determine the effect of FodHV2 on mycelial growth rate, 100 conidia of each isolate were placed in the center of a PDA plate (three replicates), and the growth area was measured daily as previously described (Lemus-Minor et al., 2018). To analyze the effect of FodHV2 on the conidiation rate in liquid medium, 200 conidia/ml of each isolate were added to 5 ml of casein-hydrolyzed medium with AZ solution (three replicates). The cultures were incubated, and the conidia were counted at different times (Lemus-Minor et al., 2018). Data, growth area and conidia⋅ml^–1^, were used to do an analysis of variance (ANOVA). Significant differences among means for daily values of growth area or conidiation obtained with each isolate were determined using Tukey’s honest significant difference (HSD; *P* ≤ 0.01) test. To determine the effect of FodHV2 on the virulence, isolates *Fod* 77Hyg^R^ or *Fod* 77Hyg^R^HV^+^ were inoculated on five different carnation cultivars, the resistant cultivar Galaxia and the susceptible cultivars Baltico, Candela, Master and Pink Bijou. Obtaining of inoculum, inoculation of carnation cuttings, and greenhouse conditions were as previously described (Gómez-Lama Cabanás et al., 2012). Plants of each cultivar treated with water were used as controls. Fusarium wilt symptoms were evaluated every 2 days for approximately 3 months using a scale of disease from 0 (no symptoms) to 5 (dead plant). Disease severity values were used to calculate the percentage of the standardized area under the disease progress curve (sAUDPC). ANOVA was used to analyze sAUDPC percentages. Significant differences among means for disease severity values with each isolate were determined using the Fisher’s least significant difference (LSD; *P* ≤ 0.05). This test of virulence was repeated two times.

At the end of each pathogenicity test, two plants inoculated with each *Fod* 77Hyg^R^ or *Fod* 77Hyg^R^HV^+^ were used to re-isolate the fungus, as described in Lemus-Minor et al. (2018). Stem sections from the selected carnation plants were placed on V8 agar plates (Komada, 1975) and incubated at 25°C in the dark. Mycelia from the fungal colonies obtained were analyzed by cellulose column chromatography to confirm presence or absence of mycovirus FodHV2.

## Results

### Detection of Viral dsRNA in *F. oxysporum* f. sp. *dianthi*

**FIGURE 1:**
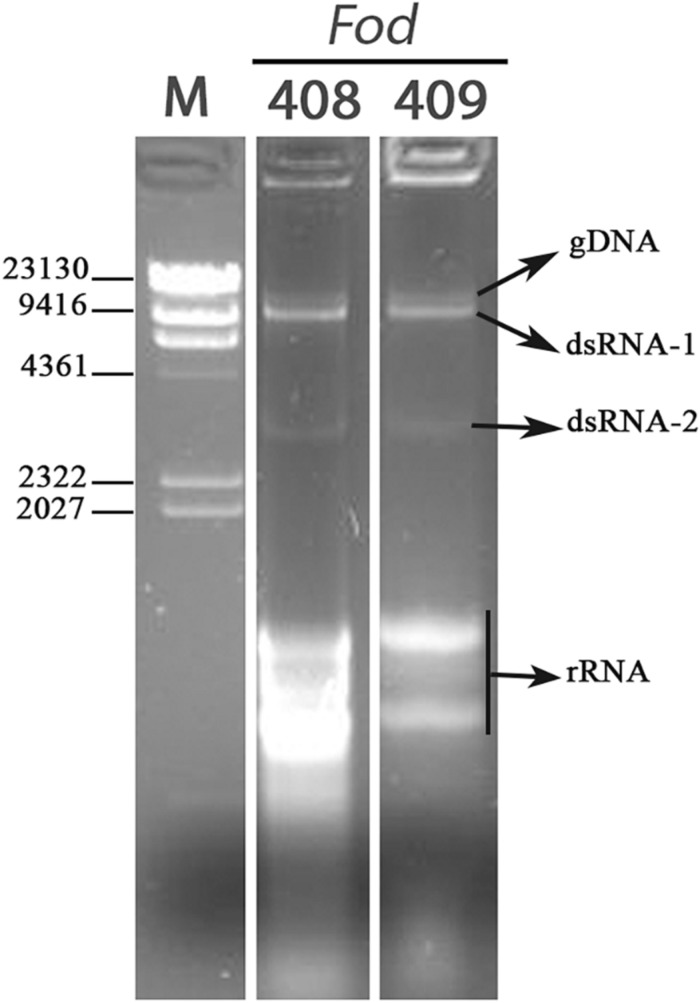
Detection of dsRNAs. Agarose gel electrophoresis of the dsRNA-enriched extracts obtained by cellulose column chromatography from isolates *Fod* 408 and *Fod* 409 of *Fusarium oxysporum* f. sp. *dianthi* from Morocco. Lane M: molecular weight marker II (Roche Diagnostics).

A total of 27 *Fusarium oxysporum* f. sp. *dianthi* (*Fod*) isolates, all of them obtained from carnation plants and soil samples from Morocco, was used in this work (Table 1). The isolates were first characterized by multiplex-PCR and determined to belong to two different races-molecular groups (Table 1), and then subjected to dsRNA purification by cellulose column chromatography. Direct observation of the dsRNA extracts showed the presence of two potentially viral dsRNA molecules in isolates *Fod* 408 and *Fod* 409 (Figure 1). The two dsRNA bands had estimated sizes of ∼9.5 kbp (dsRNA-1) and ∼3.0 kbp (dsRNA-2), respectively. Both segments were confirmed to be dsRNA in nature based on its resistance to digestion with DNase I, but susceptibility to degradation by RNase A treatment (Supplementary Figure 1). We selected isolate *Fod* 408 to characterize the dsRNA elements identified.

### Molecular Characterization of a Novel Mycovirus Infecting *F. oxysporum* f. sp. *dianthi*

A total of 96 clones were obtained and sequenced. Every region of the genome was determined by sequencing of at least five independent clones. The amplification of a specific PCR product using an oligo-dT primer demonstrated that the dsRNA molecule contained a poly(A) tail at the 3′-terminus of its positive strand. The complete genome sequence was determined to be 9,444 nt in length, excluding the poly(A) tail. Only six partial sequences were obtained for the ∼3.0 kb dsRNA-2 segment. Although these sequences showed a variable nt identity (between 41 and 81%) exclusively with the corresponding nt sequences in dsRNA-1, the exact nature of this dsRNA-2 has not been confirmed (not shown).

**FIGURE 2:**
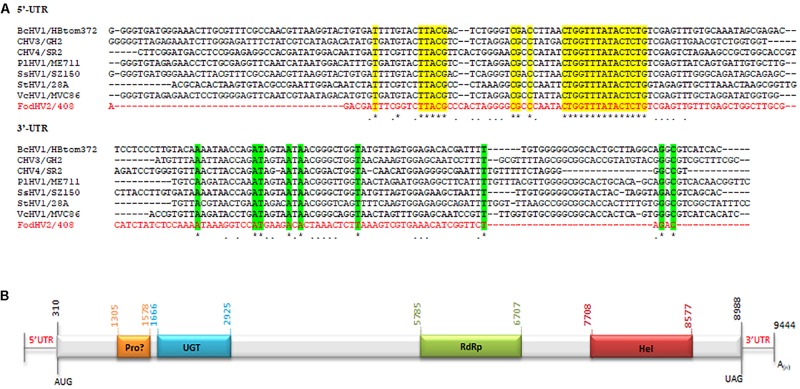
Molecular characteristics of Fusarium oxysporm f. sp. dianthi hypovirus 2 (FodHV2). **(A)** Multiple alignment of the first 100 nt of the 5′-UTR and the last 100 nt (excluding the poly(A) tail) of the 3′-UTR among FodHV2/408 and the hypoviruses BcHV1/HBtom372, CHV3/GH2, CHV4/SR2, PlHV1/ME711, SsHV1/SZ-150, StHV1/28A, and VcHV1/MVC86 (see Table 2 for complete name and accession number of the hypoviruses). Identical nt shared by FodHV2/408 and all the other hypoviruses are shown by asterisk and color shading. Identical nt in FodHV2/408 and some of the other mycoviruses are indicated by a point. Alignments were performed using CLUSTALW with the program MAFFT v7. **(B)** Schematic representation of the genomic organization of FodHV2. The genome is 9.444 bp long and contains a unique ORF that encode a possible papain-like protease (Pro), UDP glucose/sterol glucosyltransferase (UGT), RNA dependent RNA polymerase (RdRp), and helicase (Hel) domains. A poly(A) tail at the 3′-end of the coding strand is represented as A(n).

A homology search with the nt sequence using the BlastX program showed significant similarities (e-value of < 0.01) between the deduced aa sequence and the polyprotein of Cryphonectria hypovirus 4 (CHV4/SR2), Valsa ceratosperma hypovirus 1 (VcHV1/MVC86), Phomopsis longicolla hypovirus 1 (PlHV1/ME711), Cryphonectria hypovirus 3 (CHV3/GH2), Botrytis cinerea hypovirus 1 (BcHV1/HBstr-470), Setosphaeria turcica hypovirus 1 (StHV1/28A), Sclerotinia sclerotiorum hypovirus 1-A (SsHV1-A), and Sclerotinia sclerotiorum hypovirus 1 (SsHV1/SZ-150). The aa identities with these sequences ranged between 45 and 61% (Table 2). The coding sequence was flanked by two untranslated regions (UTR) of 309 nt at the 5′ end (nt 1–309) and 456 nt at the 3′ terminus (nt 8,988–9,444) excluding the poly(A) tail. The first 100 nt of the 5′-UTR and the last 100 nt of the 3′-UTR showed high identities with those of the above-cited hypoviruses (Figure 2A). This result indicated that this mycovirus could be a new member of the *Hypoviridae* family, that we tentatively named Fusarium oxysporum f. sp. dianthi hypovirus 2 (FodHV2).

**TABLE 2:** Results from BLASTX homology search with Fusarium oxysporm f. sp. dianthi hypovirus 2 (FodHV2).

Virus^a^	Acronym	Protein	Overlap	Bit score/	GenBank	Query	GenBank
		(aa size)	(aa identities%)	*e*-value	accesion no.	cover	accesión no.
Cryphonectria hypovirus 4	CHV4/SR2	Polyprotein (2848)	1444/2862 (50)	2578/0.0	AY307099	88%	AY307099
Valsa ceratosperma hypovirus 1	VcHV1/MVC86	Polyprotein (2940)	1354/2889 (47)	2505/0.0	AB690372	90%	AB690372
Phomopsis longicolla hypovirus 1	PlHV1/ME711	Polyprotein (2848)	1212/2479 (49)	2286/0.0	KF537784.1	87%	KF537784.1
Cryphonectria hypovirus 3	CHV3/GH2	Polyprotein (2874)	1198/2550 (47)	2220/0.0	AF188515	80%	AF188515
Botrytis cinerea hypovirus 1	BcHV1/HBtom372	Polyprotein (2964)	1181/2523 (47)	2155/0.0	MG554632.1	81%	MG554632.1
Setosphaeria turcica hypovirus 1	StHV1/28A	Polyprotein (2751)	1090/1785 (61)	2141/0.0	MK279474.1	77%	MK279474.1
Sclerotinia sclerotiorum hypovirus 1-A	SsHV1-A	Polyprotein (2943)	1132/2515 (45)	2115/0.0	AWY10948	80%	AWY10948
Sclerotinia sclerotiorum hypovirus 1	SsHV1/SZ-150	Polyprotein (2948)	1131/2520 (45)	2103/0.0	AEL99352	80%	AEL99352

*^*a*^The top eight distinct viruses returned by BlastX are shown.*

Sequence analysis revealed that FodHV2 has a single 8,679 nt long ORF in the positive strand, that starts with the AUG codon in the position 310 and finishes with the UAG codon in the position 8,988. This sequence encodes a polyprotein of 2,892 aa residues with a predicted molecular mass of 268 kDa. The deduced aa sequence of the polyprotein contains highly conserved domains of UDP glucose/sterol glucosyltransferase (UGT), RNA-dependent RNA polymerase (RdRp), and viral RNA helicase (Hel). In addition, conserved Cys (Cys^339^), Hys (Hys^400^), and Gly (Gly^423^) residues in the N-terminal region of the polyprotein could represent the catalytic site of a highly diverged papain-like protease (Figure 2B).

**FIGURE 3:**
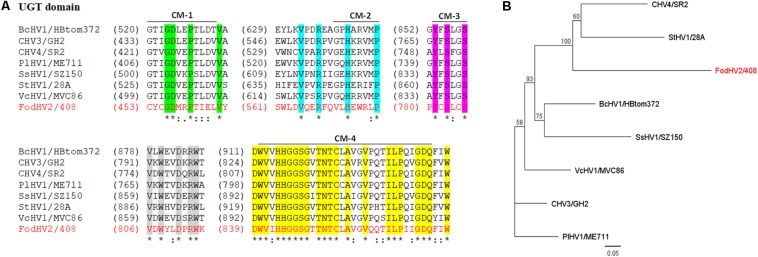
Sequence comparison and phylogenetic tree of the UGT domain of Fusarium oxysporm f. sp. dianthi hypovirus 2 (FodHV2). **(A)** Multiple alignment of the aa sequence of the UGT domain between FodHV2/408 and selected viruses in the family *Hypoviridae* [BcHV1/HBtom372, CHV3/GH2, CHV4/SR2, PlHV1/ME711, SsHV1/SZ-150, StHV1/28A, and VcHV1/MVC86 (see Table 2 for complete name and accession number of the hypoviruses)]. Identical residues are indicated with asterisk and color shading; colons and dots indicate conserved and semi-conserved aa residues. CM: conserved motif sequences (1–4) of the UDP-glucosyltransferases superfamily. The alignements were performed using CLUSTALW with the program MAFFT v7. **(B)** Phylogenetic analysis based on the aa sequence of the UGT domain of FodHV2 and the mycoviruses used for the multiple alignment. The phylogenetic tree was constructed using the program Tree View of Geneious 8.1.5 package (Biomatters), and generated by the NJ method, with 1000 bootstrap replicates.

**FIGURE 4:**
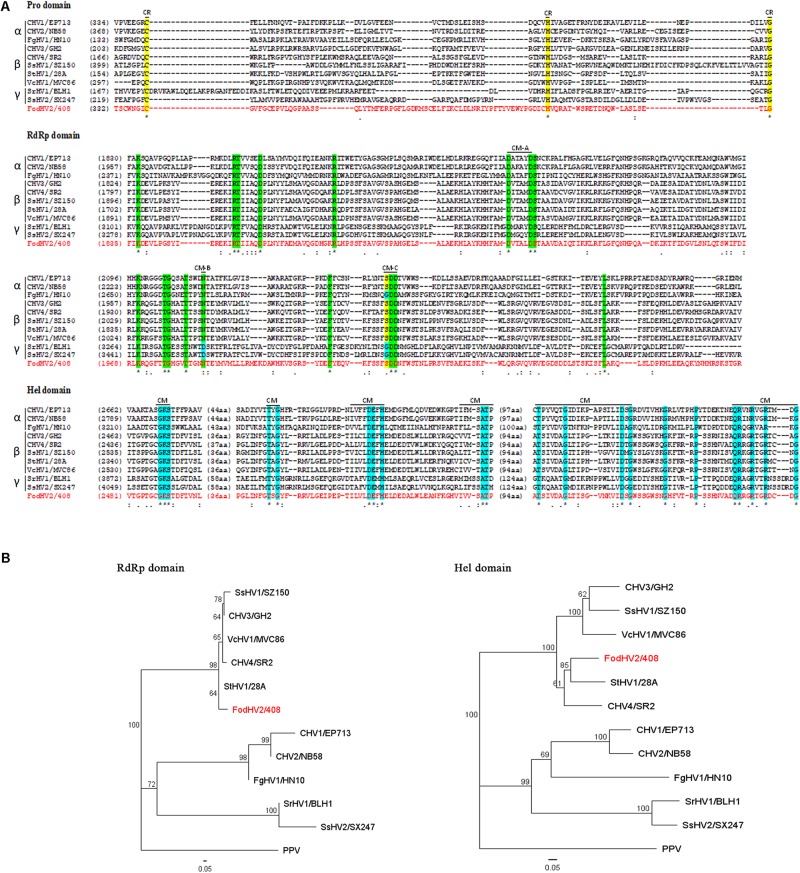
Alignments and phylogenetic trees of the Prot, RdRp, and Hel domains of Fusarium oxysporm f. sp. dianthi hypovirus 2 (FodHV2). **(A)** Multiple alignments of the aa sequences of the protease (Pro), RNA dependent RNA polimerase (RdRp), and helicase (Hel) domains of FodHV2/408 and selected viruses in the family *Hypoviridae* (see Table 3 for acronym, complete name, and GenBank accesion number of the hypoviruses used in this analysis). Alignments were performed using CLUSTALW with the program MAFFT v7. Identical residues are indicated with asterisk and color shaded; colons and dots indicate conserved and semi-conserved aa residues, respectively. Conserved residues (CR) and conserved motifs (CM) characteristic of each domain are indicated with a black line and his acronym. **(B)** Phylogenetic trees based on the RdRp and Hel aa sequences of FodHV2/408 and the selected hypoviruses. Phylogenetic trees were constructed using the program Tree View of Geneious 8.1.5 package (Biomatters), and generated by the NJ method with 1000 bootstrap replicates. Plum pox virus (PPV) was used as an out-group.

The UGT domain (nt 1,666–2,925) is 420 aa in length. Alignment of the aa sequence of this UGT domain with that of other mycoviruses in the *Hypoviridae* family identified the four highly conserved motifs (CM-1 to 4; Figure 3) as previously described (Warnecke et al., 1999). Downstream from the UGT domain (nt 5,785–6,707) a 308 aa long RdRp domain with the highly conserved motifs A, B, and C that form the catalytic center of this enzyme was identified. In particular the motif C was constituted by the SDD tripeptide, which is present in RdRps encoded by most of the *Hypoviridae* members (Hansen et al., 1997; O’Reilly and Kao, 1998; Gohara et al., 2000) (Figure 4A). Finally, a helicase domain of 290 aa was identified at the C-terminal region (nt 7708–8577). This Hel domain contained six out of the eight conserved motifs that constitute the DEAD, DEAH, and DEXH boxes characteristic of the RNA Helicases in the Superfamily II (Linder et al., 1989) (Figure 4A). This genomic organization is similar to that described for viruses belonging to the genus *Hypovirus*, suggesting that FodHV2 is a new member of the family *Hypoviridae*. The complete sequence has been deposited in the GeneBank database under the accession number MN176979.

### Phylogenetic Relationship Between FodHV2 and Other Hypoviruses

**FIGURE 5:**
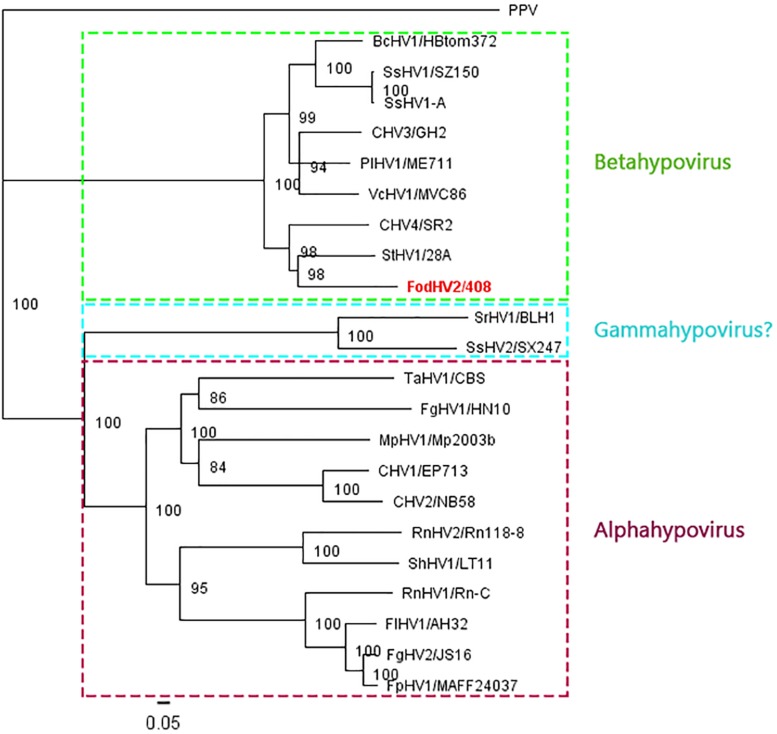
Phylogenetic analysis of Fusarium oxysporm f. sp. dianthi hypovirus 2 (FodHV2) based on the aa sequence of the polyprotein. Neighbor-Joining consensus tree of FodHV2/408 and all the other hypoviruses [Botrytis cinerea hypovirus 1 (BcHV1/HBtom372; accession no. MG554632), Sclerotinia sclerotiorum hypovirus 1 (SsHV1/SZ-150; accession no. AEL99352), Sclerotinia sclerotiorum hypovirus 1-A (SsHV1-A; accession no. AWY10948), Chryphonectria hypovirus 3 (CHV3/GH2; accession no. AF188515), Phomopsis longicolla hypovirus 1 (PlHV1/ME711; accession no. KF537784), Valsa ceratosperma hypovirus 1 (VcHV1/MVC86; accession no. AB690372), Chryphonectria hypovirus 4 (CHV4/SR2; accession no. AY307099), Setosphaeria turcica hypovirus 1 (StHV1/28A; accession no. MK279474), Sclerotium rolfsii hypovirus (SrHV1/BLH1; accession no. AZA15168), Trichoderma asperellum hypovirus 1 (TaHV1/CBS; accession no. MK279475), Sclerotinia sclerotiorum hypovirus 2 (SsHV2/SX247; accession no. AIA61616), Fusarium graminearum hypovirus 1 (FgHV1/HN10; accession no. AZT88611), Macrophomina phaseolina hypovirus 1 (MpHV1/Mp2003b; accession no. ALD89099), Chryphonectria hypovirus 1 (CHV1/EP713; accession no. M57938), Chryphonectria hypovirus 2 (CHV2/NB58; accession no. L29010), Rosellinia necatrix hypovirus 1 (RnHV1/Rn-C; accession no. PRJNA485481), Rosellinia necatrix hypovirus 2 (RnHV1/Rn118-8; accession no. BBB86776), Sclerotinia homoeocarpa hypovirus 1 (ShHV1/LT11; accession no. MK279473), Fusarium langsethiae hypovirus 1 (FlHV1/AH32; accession no. YP_009330037), Fusarium graminearum hypovirus 2 (FgHV2/JS16; accession no. PRJNA485481), Fusarium poae hypovirus 1 (FpHV1/MAFF24037; accession no. BAV56305), and the plant potyvirus Plum pox virus (PPV) as an out-group] based on the full length aa sequence of the viral polyprotein. The color boxes indicate the different proposed genera of “Alphahypovirus,” “Betahypovirus,” and “Gammahypovirus.” The phylogenetic tree was constructed using Tree View of Geneious 8.1.5 package (Biomatters), and generated by the NJ method with 1000 bootstrap replicates.

To understand the phylogenetic relationship between FodHV2 and other members in the family *Hypoviridae*, a phylogenetic analysis based on the Neighbor-Joining method was performed using the full-length aa sequence of the viral polyprotein. Result obtained distributed hypoviruses into the three distinct phylo groups corresponding to proposed genera of “Alphahypovirus,” “Betahypovirus,” and “Gammahypovirus” (Yaegashi et al., 2012; Hu et al., 2014; Khalifa and Pearson, 2014; Li et al., 2015), with FodHV2 grouping in the “Betahypovirus” clade (Figure 5). Similar topology was observed in trees constructed with aa sequences of RdRp and helicase domains (Figure 4B). Pairwise comparisons of FodHV2 nt and aa sequences with currently recognized and putative members of the family *Hypoviridae* revealed the highest homology with CHV4/SR2 and StHV1/28A (Table 3).

**TABLE 3:** Nucleotide and amino acid identities between FodHV2 and other mycoviruses in the family *Hypoviridae.*

	Virus^a^	Acronym	Full sequence		Non-coding region (nt%)^b^		Coding region (aa%)^c^				Accesion no.
			Nt%	Aa%	5′	3′	Pro	UGT	RdRp	Hel	
**α**	Cryphonectria hypovirus 1	CHV1/EP713	29	8^d^	28	25	10^e^	–^f^	14	21	M57938
	Cryphonectria hypovirus 2	CHV2/NB58	28	8^d^	28	28	11	–^f^	12	21	L29010
	Fusarium graminearum hypovirus 1	FgHV1/HN10	28	7^d^	27	27	10	–^f^	13	20	AZT88611
**β**	Cryphonectria hypovirus 3	CHV3/GH2	46	41	39	26	16	47	65	55	AF188515
	Cryphonectria hypovirus 4	CHV4/SR2	49	47	49	29	17	51	66	64	AY307099
	Sclerotinia sclerotiorum hypovirus 1	SsHV1/SZ150	44	39	28	30	14	42	64	52	JF781304
	Setosphaeria turcica hypovirus 1	StHV1/28A	48	46	50	30	10	47	72	69	JF781304
	Valsa ceratosperma hypovirus 1	VcHV1/MVC86	49	43	38	24	16	49	66	58	AB690372
**γ**	Sclerotium rolfsii hypovirus 1	SrHV1/BLH1	27	7	22	28	6	–^f^	12	14	MH037014
	Sclerotinia sclerotiorum hypovirus 2	SsHV2/SX247	24	7	28	27	10	–^f^	11	13	KJ561218

*^*a*^αβγ: selected viruses from the “Alphahypovirus,” the “Betahypovirus,” and the “Gammahypovirus” groups, respectively. ^*b*^The terminal 100 nucleotides at the 5′ and 3′-UTR were compared to the respective UTR of each virus. ^*c*^The amino acid sequence of Protease, UGT, RdRp, and Helicase domains of each hypovirus shown in Figure 4A was used for comparison. ^*d*^The ORFB polyprotein of CHV1, CHV2, and FgHV1 was used for this comparison. ^*e*^The protease domain in ORFA of CHV1 was used for comparison. ^*f*^No UGT domain was detected in the polyprotein of CHV1, CHV2, FgHV1, SrHV1 and SsHV2.*

### Incidence of FodHV2 Infections in the Collection of *F. oxysporum* f. sp. *dianthi* Isolates Analyzed

Initially, two dsRNA elements (dsRNA-1 and dsRNA-2) with comparable electrophoretic migration rates were detected in isolates *Fod* 408 and *Fod* 409 of *F. oxysporum* f. sp. *dianthi*. Characterization of the dsRNA-1 element of isolate *Fod* 408 identified it as a genome of FodHV2, a putative new member in the family *Hypoviridae*. To confirm the identity of the mycovirus detected in isolate *Fod* 409, the dsRNA purified from this isolate was subjected to RT-PCR amplification using specific primers directed to the RdRp of FodHV2. The 1.2 kb amplicon obtained was purified from the agarose gel and sequenced. Comparison of this sequence with the corresponding sequence of FodHV2 showed a 100% identity (Supplementary Figure 2), and therefore indicated that fungal isolate *Fod* 409 was also infected with hypovirus FodHV2.

Although the rest of isolates analyzed did not show any traces of viral dsRNA in the cellulose column chromatography extracts, all those extracts were also subjected to RT-PCR to discard the possibility of not-observable low-viral load FodHV2 infections. Results obtained indicated that no additional isolates were infected with FodVH2 (not shown).

### Biological Effect of FodHV2 on *F. oxysporum* f. sp. *dianthi*

**FIGURE 6:**
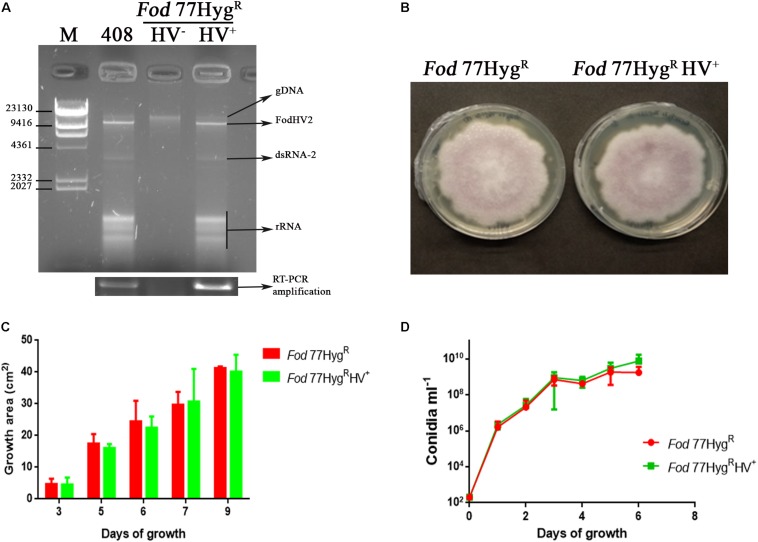
Biological effect of FodHV2 on *Fusarium oxysporum* f. sp. *dianthi*. **(A)** Agarose gel electrophoresis of the dsRNA extracts from the strains *Fod* 408 (the originally infected with FodHV2), *Fod* 77Hyg^R^HV^–^ (not infected), and *Fod* 77Hyg^R^HV^+^ (the new infected strain to which the virus was transferred), and RT-PCR products obtained using these dsRNA extracts and specific primers for the RdRp segment of FodHV2. M: molecular weight marker II (Roche Diagnostics). **(B)** Colony morphology of F*od* 77Hyg^R^ and *Fod* 77Hyg^R^HV^+^ after 9 days of growth on PDA. **(C)** Two dimensional colony growth rate of *Fod* 77Hyg^R^ and *Fod* 77Hyg^R^HV^+^. The colony area was measured at 3, 5, 7, and 9 days of growth on PDA with hygromycin at 25°C in the dark. Values are the average area of five colonies. **(D)** Conidiation rate of *Fod* 77Hyg^R^ and *Fod* 77Hyg^R^HV^+^ in liquid medium. Isolates were cultured in casein hydrolyzed medium, and conidia counted at 1, 2, 3, 4, 5, and 6 days of growth. Vertical lines in C and D represent the standard error.

In order to study the possible effect of FodHV2 on its host, we attempted obtaining two isogenic isolates infected and not-infected with FodHV2. In a first attempt, we tried to obtain a virus-free line of the naturally infected isolate *Fod* 408 by single conidia selection. A total of 40 monoconidial cultures were analyzed by cellulose column chromatography. Agarose gel electrophoresis of the dsRNA extracts showed that all monoconidial cultures were infected with FodHV2 (Supplementary Figure 3). Alternatively, the obtention of isogenic virus-free and virus-infected strains was achieved by passing mycovirus FodHV2 through hyphal anastomosis between the strain originally infected (*Fod* 408, donor) and another Hyg^R^*-*tagged virus-free strain (*Fod* 77Hyg^R^, recipient). As a result, two isogenic strains, not infected (*Fod* 77Hyg^R^) and infected (*Fod* 77Hyg^R^HV^+^) with mycovirus FodHV2, were obtained (Figure 6).

Effect of FodHV2 on host growth on solid medium and conidiation rate in liquid medium was estimated by comparison of both *Fod* 77Hyg^R^ and *Fod* 77Hyg^R^HV^+^ isolates. Results obtained showed that neither the morphology and radial growth rate on solid medium, nor the conidiation rate in liquid medium, were significantly affected by the FodVH2- infection (Figure 6).

To determine the effect of FodHV2 on virulence, two pathogenicity tests were performed in which several susceptible carnation cultivars were inoculated with each *Fod* 77Hyg^R^ and *Fod* 77Hyg^R^HV^+^ strains. Factorial analysis of the disease data showed that there were no significant differences (*P* ≤ 0.05) between the sAUDPC percentages obtained with all the susceptible carnation cultivars and the two inoculated strains (Table 4). Fungal isolation assays performed with representatives of plants inoculated with either *Fod* 77Hyg^R^ or *Fod* 77Hyg^R^HV^+^ showed in both cases an extensive colonization of the plants vascular-system (Supplementary Figure 4). Purification of the dsRNA extracts obtained by cellulose column chromatography from the re-isolated colonies confirmed the presence of FodHV2 in isolate *Fod* 77Hyg^R^HV^+^, and its absence in the case of isolate *Fod* 77Hyg^R^ (Supplementary Figure 4).

**TABLE 4:** Percentage of the standardized area under the disease progress curve (sAUDPC)^*y*^.

		Carnation cultivar				
Test date	Isolate inoculated	Susceptible				Resistant
		Candela	Baltico	Master	Pink Bijou	Galaxia
June 2016	*Fod* 77 Hyg^R^	0.882^a^	0.772^b^	0.682^c^	0.977^d^	0
	*Fod* 77 Hyg^R^HV^+^	0.887^a^	0.735^b^	0.700^c^	0.990^d^	0
April 2017	*Fod* 77 Hyg^R^	0.427^a^	0.417^ab^	0.533^bc^	0.848^d^	0
	*Fod* 77 Hyg^R^HV^+^	0.328^a^	0.552^b^	0.551^bc^	0.848^d^	0

*^*y*^Means followed by the same letter in each test date are not significantly different according to Fisher’s least significant difference (LSD) (*P* ≤ 0.05).*

## Discussion

In this study we have molecularly and biologically characterized a novel mycovirus identified in isolate *Fod* 408 of *Fusarium oxysporum* f. sp. *dianthi*. The complete viral genome is 9,444 nt long, excluding the poly(A) tail, and comprises a 309-nt 5′-UTR, an 8,679-nt single ORF encoding a polyprotein of 2,892 aa residues and 268 kDa, and a 456-nt 3′-UTR. The polyprotein contains three highly conserved domains of UGT, RdRp, and helicase. In addition, identification of conserved Cys, Hys, and Gly residues in the N-terminal region of the polyprotein suggests the presence of a catalytic site of a highly diverged protease. Homology searches revealed a high sequence similarity with the polyprotein of other mycoviruses in the genus *Hypovirus*; this similarity extended also to the 5′- and 3′-UTR regions. Based on these results, we propose that this virus is a novel member of the family *Hypoviridae*, for which we propose the name Fusarium oxysporum f. sp. dianthi hypovirus 2 (FodHV2).

Members of all four currently recognized species in the genus *Hypovirus* were isolated from *C. parasitica* (Hillman et al., 1990, 1992, 1994; Enebak et al., 1994; Smart et al., 1999; Linder-Basso et al., 2005; Nuss and Hillman, 2011), but a number of unclassified hypovirus-like viruses have been described infecting other filamentous fungi, e.g., *Agaricus bisporus* (Deakin et al., 2017), *Alternaria alternata* (Li et al., 2019), *Macrophomina phaseolina* (Marzano et al., 2016), *Phomopsis longicolla* (Koloniuk et al., 2014), *Sclerotinia sclerotiorum* (Xie et al., 2011; Hu et al., 2014; Khalifa and Pearson, 2014; Marzano et al., 2015), and *Valsa ceratosperma* (Yaegashi et al., 2012). The only four possible members of the family *Hypoviridae* described from *Fusarium* species are FgHV1 and FgHV2 from *F. graminearum* (Wang et al., 2013; Li et al., 2015), FlHV1 from *F. langsethiae* (Li et al., 2017), and FpHV2 from *F. poae* (Osaki et al., 2016). In this work we have detected FodHV2 in a strain of *F. oxysporum* f. sp. *dianthi*. Therefore, FodHV2 is the first hypovirus described infecting the important plant pathogenic species *F. oxysporum.* Another unique feature of FodVH2 is that it has been detected infecting isolate *Fod* 408 obtained from a symptomatic carnation plant sampled in Morocco. Therefore, it is the first hypovirus described in the African continent.

Based on differences in the genomic organization and on results from phylogenetic analyses, Yaegashi et al. (2012) proposed the distinction of two genera within the family *Hypoviridae*: the genus “Alphahypovirus,” represented by CHV1 and CHV2, and the genus “Betahypovirus,” represented by CHV3 and CHV4. The successive description of newly proposed members in this family has supported this reorganization of the family, since the majority of the novel mycoviruses identified could be assigned to either of the two proposed genera. Exceptions are Sclerotinia sclerotium hypovirus 2 (SsHV2, isolates 5472 and SX247) and Sclerotium rolfsii hypovirus 1 (SrHV1/AZA15168), the only three hypoviruses that do not fit in the “Alpha-” or “Beta-hypovirus” genera, and for which a creation of a new genus (“Gammahypovirus”) has been recently proposed (Hu et al., 2014). Genome length, genomic organization, and phylogenetic analysis, clearly indicate that FodHV2 could be assigned to the “Betahypovirus” group. As in other possible members in that putative genus, genome of FodHV2 is shorter than that of the members in the Alpha- or Gamma- groups, and contains a single ORF. The encoded polyprotein includes protease, RdRp, and helicase domains, characteristic of all hypoviruses, but also contains a distinctive UGT domain which is encoded solely by the members of the “Betahypovirus” group (Yaegashi et al., 2012; Gilbert et al., 2019). In addition, viruses in the putative genus “Betahypovirus” share sequence conservation in the 5′-UTR, including a 15-nt sequence (CTGGTTTATACTCTG) present in all known putative betahypoviruses (Gilbert et al., 2019). The comparison of the complete nt and aa sequences between FodHV2 and other mycoviruses in the family *Hypoviridae* showed the greatest similarity with the betahypoviruses CHV4 (49 and 47%, respectively), and StHV1 (48 and 46%, respectively). Similar results were obtained when single functional domains were used for comparisons. Multiple alignments of the UGT, RdRp, and Hel domains between FodHV2 and other hypoviruses, allowed the identification of a number of conserved motifs. Alignments for the UGT aa sequence identified the four conserved motifs (CM-1 to 4) characteristic of UDP-glucosyltransferases (Warnecke et al., 1999) which are present in betahypoviruses, but not in alpha- or gammahypoviruses. Multiple alignments of the RdRp domain identified the motifs A (D—D), B (N), and C (SDD/GDD) characteristic of viral RdRps (Hansen et al., 1997; O’Reilly and Kao, 1998; Gohara et al., 2000). Particularly, the presence of tripeptide SDD constituting “motif C” has been described for most of the *Hypoviridae* members. Interestingly, the presence of a D residue instead of an N residue in motif B seems to differentiate gammahypoviruses from alpha- or beta-hypoviruses. Finally, multiple alignments of the Hel domain identified six out of the eight conserved motifs representative of the DEAD, DEAH and DEXH boxes, which are characteristic of RNA helicases in the Superfamily II (Linder et al., 1989). As previously noted with other hypoviruses (Yaegashi et al., 2012), homology among the Prot domain showed to be lower (between 6 and 17%) than those obtained with the UGT, RdRp, or Hel domains. Contrary to the alphahypoviruses that contain active proteases in each ORF, members of the “Betahypovirus” group seem not to have an active protease, because only the catalytic residues of Cys, Hys, and Gly could be identified in the alignments (Koonin et al., 1991; Hillman et al., 1994; Smart et al., 1999). Phylogenetic analyses based on the full-length aa sequence of the viral polyprotein, or on the sequence of each RdRp, and Hel protein domains, all clustered FodHV2 with the members of the “Betahypovirus” group.

In order to analyze the putative effect on its fungal host, we transferred FodHV2 from the original infected strain *Fod* 408 (donor) to another virus-free strain (recipient) by hyphal anastomosis (horizontal transmission). Strain *Fod* 77 was selected as a recipient in the first instance taking advantage of the fact that it was already transformed with a hygromicin resistance gene (*Fod* 77Hyg^R^) (Lemus-Minor et al., 2019). However, strains *Fod* 408 and *Fod* 77 had been molecularly identified as a race 2 molecular group II isolate (this work), and a race 2 molecular group I isolate (Gómez-Lama Cabanás et al., 2012), respectively. As previously determined (Gómez-Lama Cabanás and Pérez-Artés, 2014), *F. oxysporum* f sp. *dianthi* isolates of these two race-molecular groups belong to different vegetative compatibility groups (VCGs) and therefore should be vegetatively incompatible. Despite the expected incompatibility, FodHV2 was successfully transferred from isolate *Fod* 408 to isolate *Fod* 77Hyg^R^. Additionally, many of the *Fod* 77Hyg^R^ colonies analyzed for the presence of FodHV2 also showed the presence of the dsRNA-2 segment, although the accumulation of this dsRNA varied notably among colonies. In most filamentous fungi, the heterogenic incompatibility acts as a barrier to prevent the spread of mycoviruses (Caten, 1972; Esser, 2006), but mycoviruses may occasionally escape from this barrier system. For example, in *C. parasitica* some of the hypoviruses could be transmitted in particular incompatible combinations (Cortesi et al., 2001; Biella et al., 2002; Choi et al., 2012), which suggests that the timing or strength of the programed cell death associated to heterogenic incompatibility can be different for different combinations of *vic* (vegetative incompatible) genes. Moreover, the compatibility between isolates belonging to different VCGs has been described for different *formae speciales* of *F. oxysporum*, including *F. oxysporum* f. sp. *dianthi* (Gómez-Lama Cabanás and Pérez-Artés, 2014). Fungal isolates showing compatibility with others that belong to different VCG can be considered as “bridge” isolates, and might represent intermediates toward the formation of “new” VCGs (Katan et al., 1991; Cai et al., 2003). Taking this into account, isolates *Fod* 408 and/or *Fod* 77 could be considered one of these “bridge” isolates. This hypothesis would be supported by the fact that FodHV2 has also been detected infecting isolate *Fod* 409, another isolate from Morocco that, interestingly, belongs to a different race and VCG than *Fod* 408.

Along with the facility to transfer horizontally, the transmission of FodHV2 through the conidia (vertical transmission) proved highly efficient. During the initial attempt to obtain a virus free representative of isolate *Fod* 408 by single conidia selection, we obtained a total of 40 single-conidia cultures that were all infected with FodHV2. In addition, a similar analysis with the new infected isolate *Fod* 77Hyg^R^HV^+^ also showed 100% efficiency in the vertical transmission of the virus.

Comparison of isogenic strains *Fod* 77Hyg^R^ (not infected) and *Fod* 77Hyg^R^HV^+^ (infected) showed that FodHV2 did not affect the vegetative growth, the conidiation, or the virulence of the *F. oxysporum* f. sp. *dianthi* infected strain. Additionally, results from fungal isolation assays performed with representatives of inoculated carnation plants indicated that both the virus-free *Fod* 77Hyg^R^ and the virus-infected 77Hyg^R^HV^+^ isolates had a similar capacity to colonize the vascular system of the plant. Mycoviruses in the family *Hypoviridae* were initially associated with the induction of hypovirulence and/or the alteration of the vegetative growth in their hosts, but successive identification of new representatives in this family evidenced a cryptic nature in some of them. The “Alphahypovirus” and “Betahypovirus” proposed genera in that family include both viruses that alter phenotypic characters including virulence, and viruses with no apparent effect on the host. In this regard, it is interesting to mention that hypoviruses CHV4/SR2 and VcHV1/MVC86, two of the most molecularly similar to FodHV2, are also viruses with no visible effect on its fungal host (Linder-Basso et al., 2005; Yaegashi et al., 2012).

Along with the FodHV2 dsRNA, the presence of another shorter molecule was detected in isolate *Fod* 408. Partial sequence analysis of that dsRNA-2 showed a variable nt identity exclusively with the corresponding nt sequences of FodHV2, but the exact nature of this dsRNA has not been confirmed. The presence of both the complete hypovirus-dsRNA and another, possibly defective-dsRNA has been described for other betahypoviruses, e.g., AaHV1 (Li et al., 2019), BcHV1 (Hao et al., 2018), CHV3/GH2 (Smart et al., 1999), and SsHV1/SZ150 (Xie et al., 2011). In some cases (SsHV1/SZ150), data obtained suggested a contribution of the defective dsRNA to the induction of hypovirulence, but not in others (AaHV1, BcHV1). In the case of FodHV2, the joint presence of both dsRNAs had no effect on mycelial growth, conidiation, or virulence. Nevertheless, more work is needed to determine the exact nature of the dsRNA-2 molecule and its possible interference (positive or negative) on the potential effect of FodHV2 on its host.

## Data Availability Statement

The datasets generated for this study can be found in the NCBI, GenBank, Accession No. MN176979.

## Author Contributions

EP-A, MC, MG-P, and AT-T conceived and designed the experiments. AT-T performed the sequencing, assembly, analysis of the sequence, phylogenetic analyses, and phenotypic analysis. MC and MG-P obtained the Hyg^R^-tagged *Fod* strain. EP-A and AT-T analyzed the data and wrote the manuscript.

## Conflict of Interest

The authors declare that the research was conducted in the absence of any commercial or financial relationships that could be construed as a potential conflict of interest.
