# Supplementary material for: Molecular and Biological Characterization of the First Hypovirus Identified in Fusarium oxysporum
Source: Front Microbiol. 2020 Jan 24;10:3131. doi: 10.3389/fmicb.2019.03131 (PMC6992542; doi:10.3389/fmicb.2019.03131)
Supplement: Supplementary file 1 [file Data_Sheet_1.PDF]

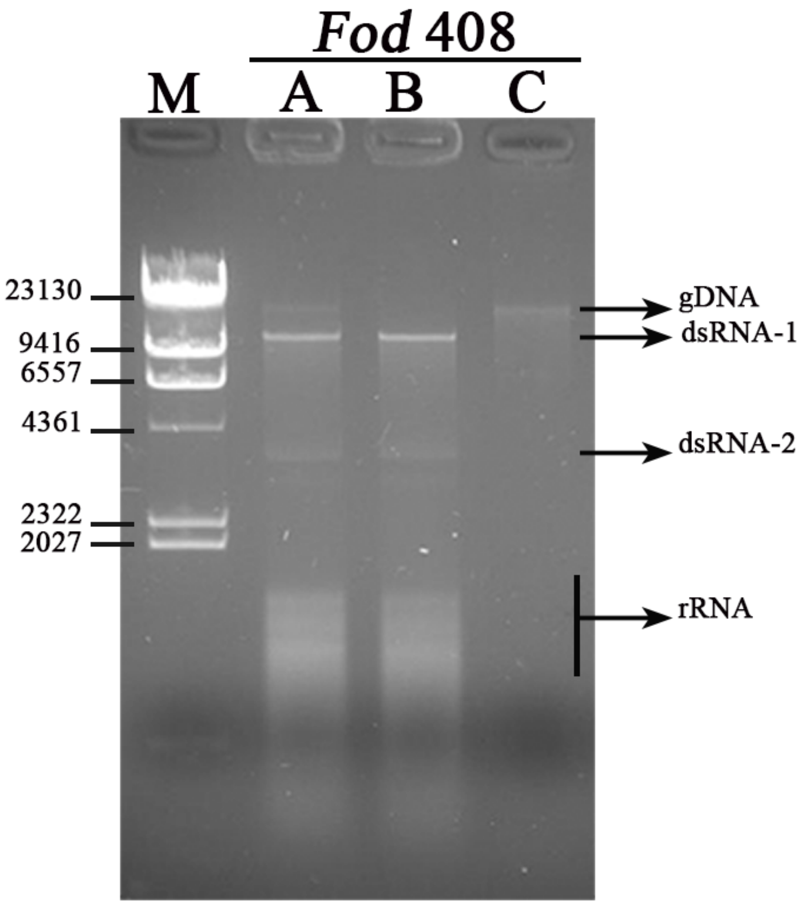

SUPPLEMENTARY FIGURE 1 | DNase I and RNase A treatment of the dsRNA-enriched extracts obtained by cellulose column chromatography. Agarose gel electrophoresis of the dsRNA-enriched extracts obtained from isolate *Fod* 408: (A) without treatment, (B) digested with DNase I, and (C) digested with RNase A at low salt conditions. Lane M: molecular weight marker II (Roche Diagnostics).
