# Supplementary material for: Molecular and Biological Characterization of the First Hypovirus Identified in Fusarium oxysporum
Source: Front Microbiol. 2020 Jan 24;10:3131. doi: 10.3389/fmicb.2019.03131 (PMC6992542; doi:10.3389/fmicb.2019.03131)
Supplement: Supplementary file 3 [file Data_Sheet_3.PDF]

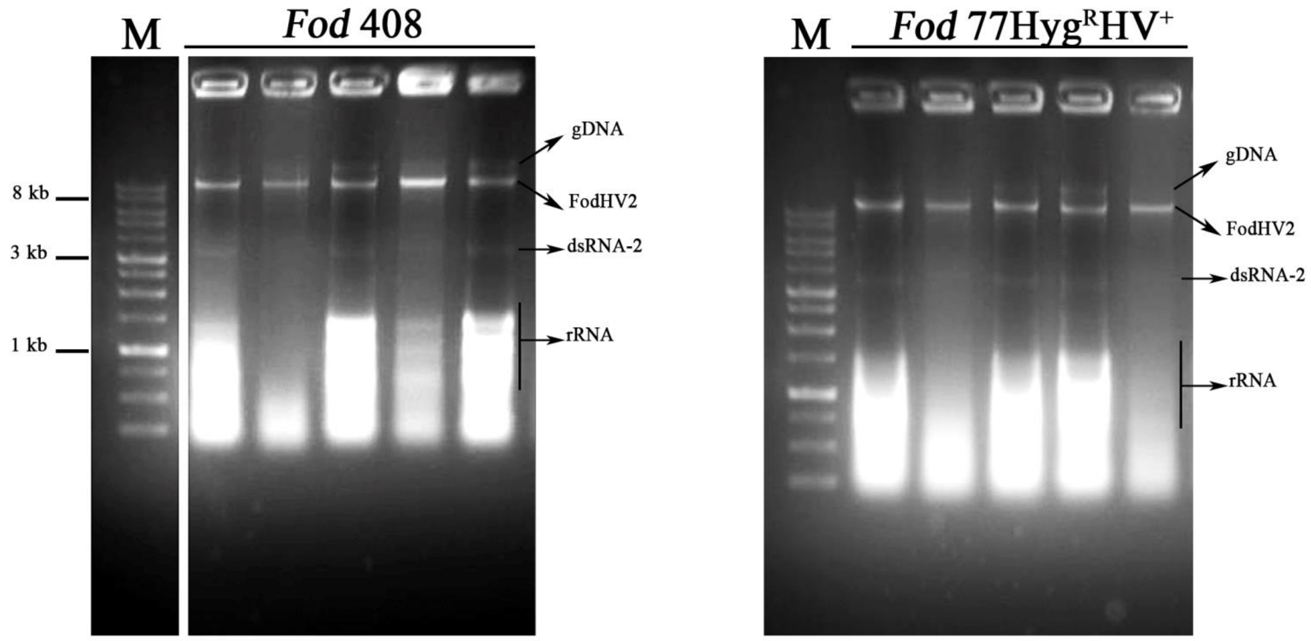

SUPPLEMENTARY FIGURE 3 | dsRNA-enriched extracts from different monoconidial cultures of the FodHV2-infected isolates *Fod* 408 and *Fod* 77Hyg^{R}HV^{+}. Agarose gel electrophoresis of the dsRNA extracts obtained by cellulose column chromatography from different monoconidial cultures obtained from the virus-infected isolates *Fod* 408 (the originally infected strain), and *Fod* 77Hyg^{R}HV^{+} (the strain to which the virus was transferred). M: 1kb molecular weight marker (Nippon Genetics).
