# Supplementary material for: Molecular and Biological Characterization of the First Hypovirus Identified in Fusarium oxysporum
Source: Front Microbiol. 2020 Jan 24;10:3131. doi: 10.3389/fmicb.2019.03131 (PMC6992542; doi:10.3389/fmicb.2019.03131)
Supplement: Supplementary file 4 [file Data_Sheet_4.PDF]

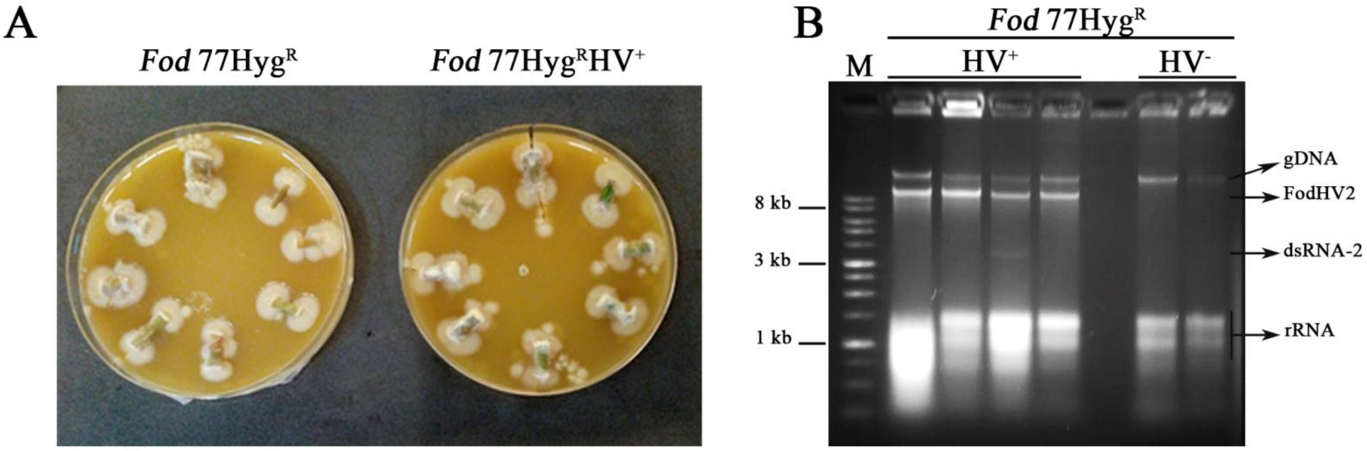

SUPPLEMENTARY FIGURE 4 | Fungal isolation from inoculated carnation plants. (A) Stem sections from carnation plants inoculated with isolate *Fod* 77Hyg^R or *Fod* 77Hyg^{R}HV$^+$ cultured on V8 agar plates. (B) Agarose gel electrophoresis of the dsRNA-enriched extracts obtained from the fungal colonies recovered showing presence (*Fod* 77Hyg^{R}HV$^+$) or absence (*Fod* 77Hyg^{R}HV$^-$) of FodHV2-dsRNA. M: 1kb molecular weight marker (Nippon Genetics).
