# Supplementary material for: Molecular and Biological Characterization of the First Hypovirus Identified in Fusarium oxysporum
Source: Front Microbiol. 2020 Jan 24;10:3131. doi: 10.3389/fmicb.2019.03131 (PMC6992542; doi:10.3389/fmicb.2019.03131)
Supplement: Supplementary file 5 [file Data_Sheet_5.PDF]

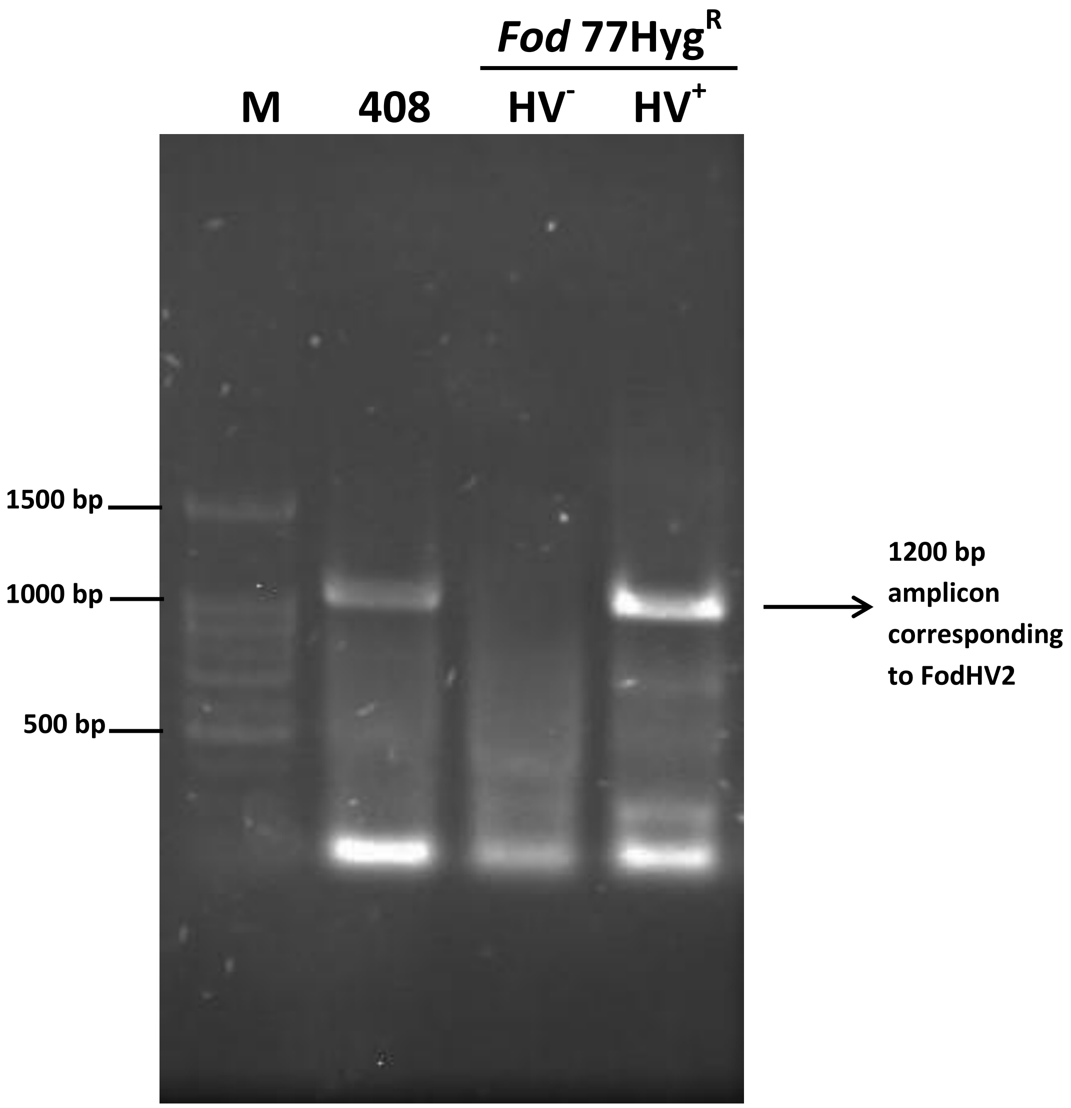

SUPPLEMENTARY IMAGE TO FIGURE 6 | Complete image corresponding to the agarose gel where the results of the RT-PCR using specific primers for the RdRp of FodHV2 are shown. RT-PCR products obtained with dsRNA extracts from isolate *Fod* 408 (the originally infected), *Fod* 77Hyg^{R}HV^{-} (not infected) and *Fod* 77Hyg^{R}HV^{+} (the new infected strain to which the virus was transferred). **M**, 100 bp molecular weight marker (Promega).
