# Supplementary material for: Molecular and Biological Characterization of the First Hypovirus Identified in Fusarium oxysporum
Source: Front Microbiol. 2020 Jan 24;10:3131. doi: 10.3389/fmicb.2019.03131 (PMC6992542; doi:10.3389/fmicb.2019.03131)
Supplement: Supplementary file 2 [file Data_Sheet_2.PDF]

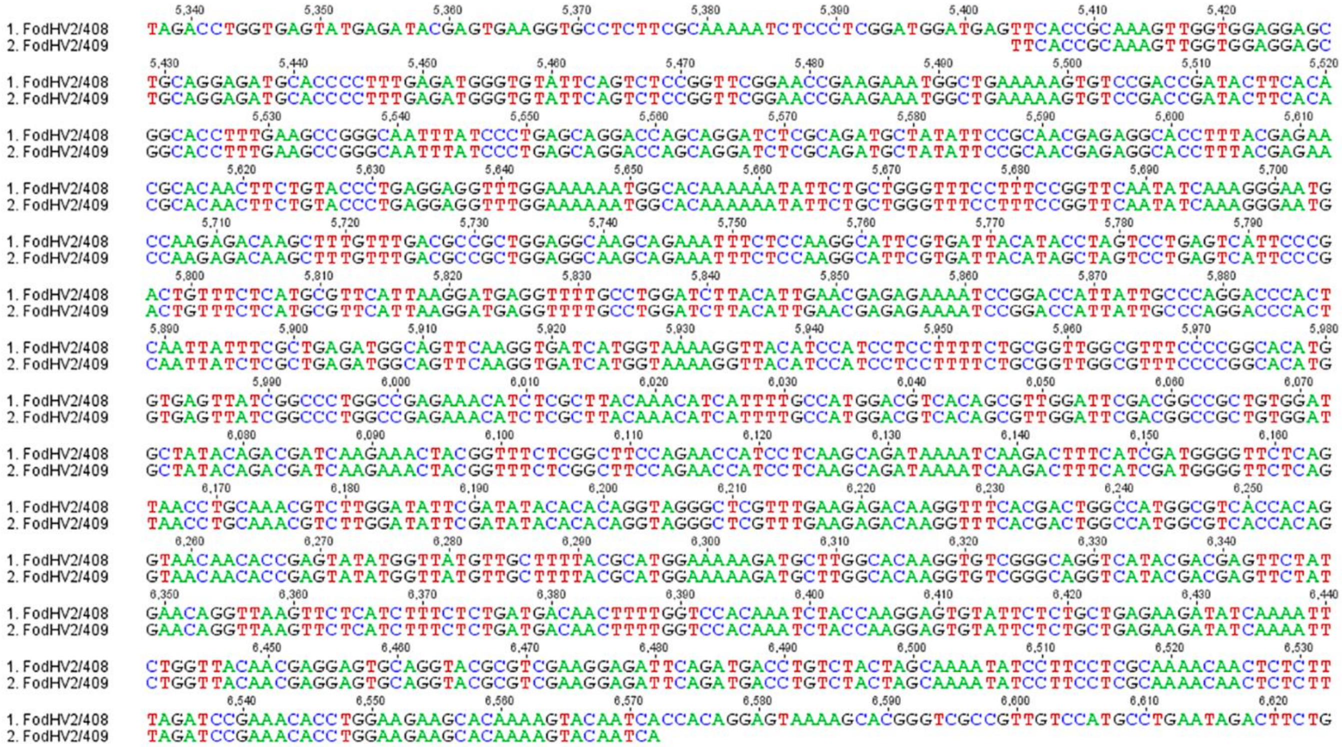

SUPPLEMENTARY FIGURE 2 | Alignment of the partial nucleotide sequence of the RdRp of FodHV2/408 and FodHV2/409. Nucleotide sequence alignment of the amplicon obtained by RT-PCR using dsRNA extracts from isolates *Fod* 408 and *Fod* 409 of *Fusarium oxysporum* f. sp. *dianthi* and specific primers directed to the RdRp of FodVH2. Analysis was carried out with the program Geneious version 8.1.5 (Biomatters).
